# Alpha-2 receptor agonists for the treatment of posttraumatic stress disorder

**DOI:** 10.7573/dic.212286

**Published:** 2015-08-14

**Authors:** Molly R Belkin, Thomas L Schwartz

**Affiliations:** SUNY Upstate Medical University, College of Medicine, Syracuse, NY, USA

**Keywords:** Adrenergic alpha-2 receptor agonists, anxiety disorders, clonidine, guanfacine, humans, stress disorders, posttraumatic, sympathetic nervous system, treatment outcome

## Abstract

Clonidine and guanfacine are alpha-2 receptor agonists that decrease sympathetic outflow from the central nervous system. Posttraumatic stress disorder (PTSD) is an anxiety disorder that is theorized to be related to a hyperactive sympathetic nervous system. Currently, the only US Food and Drug Administration (FDA)-approved medications for PTSD are the selective serotonin reuptake inhibitors (SSRIs) sertraline and paroxetine. Sometimes use of the SSRIs may not lead to full remission and symptoms of hyperarousal often persist. This article specifically reviews the literature on alpha-2 receptor agonist use for the treatment of PTSD and concludes that while the evidence base is limited, these agents might be considered useful when SSRIs fail to treat symptoms of agitation and hyperarousal in patients with PTSD.

## Introduction

Posttraumatic stress disorder (PTSD) is a psychiatric condition that develops after exposure to a trauma in the form of a threatened or actual harm, death, or sexual violence. Symptoms are divided into four clusters. The first describes intrusion symptoms, such as nightmares, flashbacks, and unwanted memories of the traumatic event. The second is defined by avoidance of places, people, or situations that trigger distressing thoughts about the traumatic event. The third includes negative alterations in cognition and mood, which may manifest as memory loss regarding the traumatic event; distorted and negative view of oneself, others, and/or the world; persistent negative mood; feelings of detachment; or anhedonia. The last cluster is defined by alterations in arousal and reactivity, such as increased irritability, anger, recklessness, hypervigilance, exaggerated startle response, decreased concentration, or sleeping difficulty [1].

It has been estimated that the prevalence of PTSD in the United States ranges from 6% to 9% [2]. However, the prevalence has been shown to be higher among populations that are at higher risk for exposure to traumatic events, such as military, fire, or police personnel; victims of sexual assault; and victims of natural disasters. Prevalence rates among these at-risk populations may be as high as 40% [2].

PTSD is often comorbid with other psychiatric disorders, most commonly major depressive disorder (MDD), alcohol use disorder (AUD), and DSM-5 anxiety disorders [2]. Substance misuse is common among those with PTSD and may serve as a means to self-alleviate symptoms. Given the association between PTSD and substance misuse, it may be reasonable to limit the use of potentially addictive benzodiazepines (BZDs) among this population and to search for other nonaddictive agents that may ameliorate the activating and hyperarousal symptoms of PTSD.

Hyperarousal symptoms may be common residual PTSD symptoms not initially treated by approved selective serotonin reuptake inhibitor (SSRI) use. Despite a conflicting evidence base, atypical antipsychotics are often used for their antihistaminergic properties to induce sedation and somnolence in patients with these residual symptoms [3,4]. Also, at higher doses, dopamine-2 receptor antagonism may theoretically improve symptoms of dysphoric or agitation. The atypicals do not risk addiction but may cause tardive dyskinesia (TD), extrapyramidal symptoms (EPS), or metabolic syndrome [5]. The antihypertensive alpha-1 receptor antagonist prazosin can also be sedating but seems to have the greatest benefit in reducing PTSD-associated nightmares [6] more so than improving daytime hyperarousal. The noradrenergic alpha-2 receptor agonists are used to dampen noradrenergic tone when used as antihypertensives. Interestingly, these agents seem to be also utilized in patients with PTSD and other psychiatric disorders to help lower agitation [7]. This class may be another off-label, nonaddictive alternative to BZD use in treating agitation, hyperarousal, and insomnia associated with PTSD.

The only Food and Drug Administration (FDA)-approved pharmacotherapy for the treatment of PTSD are the SSRIs, specifically sertraline and paroxetine. These agents may allow for a 50% symptom response compared with placebo, which affords mild to modest treatment effect sizes [8–10]. While SSRIs are the mainstay of initial PTSD pharmacotherapy, there are many other medications that are used off-label at times. The American Psychiatric Association (APA) Practice Guideline for the Treatment of Patients with Acute Stress Disorder and Posttraumatic Stress Disorder recommends SSRIs as first-line treatment and names tricyclic antidepressants (TCAs) and monoamine oxidase inhibitors (MAOIs) as acceptable alternatives [11]. The guideline also lists BZDs as an acceptable treatment for sleep and anxiety symptoms but cautions against their use due to the potential for addiction, as well as worsening of PTSD symptoms upon withdrawal. Second-generation (atypical) antipsychotics, anticonvulsants, alpha-2 receptor agonists, and beta-adrenergic blockers are also listed as acceptable options to be considered for specific target-symptom resolution. Furthermore, the noradrenergic alpha-1 receptor antagonists have gained much popularity, an expanding evidence base, and more regular clinical use in reducing PTSD nightmares specifically [12]. Although the alpha-2 receptor agonist agents seem to be gaining in their clinical use, there continues to be very little evidence base to support this practice. This article will attempt to delineate the rationale for noradrenergic dampening by use of the alpha-2 receptor agonists as adjuncts to SSRIs in the treatment of PTSD and will review the sparse, but available, literature.

## Alpha-2 receptor agonists—rationale for use in PTSD

PTSD is theorized to be related to a hyperactive sympathetic nervous system (SNS) that is a consequence of the brain’s reaction to a serious trauma. It has been observed that combat veterans with PTSD mount an exaggerated sympathetic response, as measured by heart rate, blood pressure, and sweating, when exposed to combat-related triggers [13]. Norepinephrine (NE), a key neurotransmitter of the SNS, plays a major role in this process and has been found to be linked to arousal, attention, and vigilance [14]. Not surprisingly, numerous clinical studies have shown that individuals with PTSD demonstrate increased noradrenergic response to stimuli [15]. One such study found that combat-related auditory stimuli resulted in a 30% increase in plasma NE in those with PTSD (*p*<0.001), while the same stimuli lead to no significant change in plasma NE in healthy controls [16]. An increase in baseline noradrenergic tone has also been observed in patients with PTSD. Geracioti et al. found that even in the absence of stressful stimuli, the concentration of NE in the cerebrospinal fluid (CSF) was significantly higher in patients with PTSD as compared with healthy controls. They also found that the level of CSF NE was directly related to severity of current PTSD symptoms, as measured with the Clinician-Administered PTSD Scale (CAPS) [17].

This observed elevation in noradrenergic tone in patients with PTSD provides a rationale for the use of pharmacologic SNS dampeners as a potential treatment for certain symptoms of PTSD. Central nervous system (CNS) alpha-2 adrenergic receptors function as postsynaptic autoreceptors that inhibit further sympathetic activity in the brain [15]. Both clonidine and guanfacine are alpha-2 receptor agonists that stimulate these postsynaptic adrenergic autoreceptors, ultimately decreasing SNS outflow from the CNS. This mechanism affords a lowering of blood pressure in hypertensive patients and is typically where these agents are used in medical practice. More recently, these agents have been approved in their extended-release preparations for use in childhood attention deficit hyperactivity disorder (ADHD). In this population, the alpha-2 receptors are agonized where they reside as presynaptic heteroreceptors on fronto-cortical glutamatergic neurons to improve frontal lobe functioning and efficiency [18–19]. Although there are no alpha-2 receptor agonists that are FDA-approved for the treatment of PTSD, clinicians appear to use clonidine as an adjunct for residual hyperarousal symptoms in patients with PTSD due to its known ability to lower noradrenergic activity [7].

## Alpha-2 receptor agonists—efficacy and safety in PTSD

While there have been no large, randomized, double-blind, placebo-controlled trials for the use of clonidine in the treatment of PTSD, the treatment has been observed to be effective in clinical practice. Kinzie and Leung observed that 43 of 68 Cambodian refugees with both PTSD and depression experienced symptomatic improvement with a combination therapy of imipramine (a TCA) and clonidine. With these results, Kinzie and Leung designed a prospective pilot study of nine Cambodian refugees who met the DSM-III-R [20] criteria for both chronic PTSD and MDD. They found that after 12–19 months, this combination of imipramine and clonidine resulted in a reduction of PTSD symptoms in six out of the nine patients. Two improved significantly and were deemed to be in remission from PTSD [21].

Although clonidine seems to be more widely used in clinical practice for the treatment of hyperarousal symptoms in PTSD, there have been several more recent trials investigating the use of guanfacine for these symptoms. Guanfacine is an alpha-2 receptor agonist but has greater specificity for the 2a subreceptor compared with clonidine. Clonidine actually binds to alpha-2a, 2b, and 2c receptors, which is believed to be the cause of its more sedating properties. Guanfacine selectively binds to alpha-2a receptors [19].

In a trial by Neylan et al. (n=63), guanfacine was compared with placebo using a double-blind, randomized-controlled trial of veterans with chronic PTSD. Those included were either taking no medications or following a stable pharmacotherapy regimen. All subjects met full DSM-IV [22] criteria for PTSD. Those assigned to the guanfacine group initially received 0.5 mg of guanfacine with a weekly 0.5 mg increase up to a dose of 1.0–3.0 mg over 8 weeks. The primary outcome measures were the CAPS, the Impact of Event Scale-Revised (IES-R), the Symptom Checklist-90-Revised (SCL-90-R), Sleep Quality Index, and Quality of Life Inventory. At the end of the 8-week study, Neylan et al. found that there was no significant improvement in PTSD symptoms with the use of guanfacine. They did find as a negative, however, that guanfacine was associated with a significant increase in side effects, including dry mouth and light-headedness [23].

In a 2008 placebo-controlled trial, Davis et al. compared guanfacine with placebo for the treatment of PTSD in a sample of 36 mostly-male combat veterans with PTSD. Of note, the majority of subjects was already receiving treatment with an SSRI and was considered to be treatment resistant. Davis et al. found that there was no significant improvement of PTSD symptoms or self-reported quality of life with the addition of guanfacine. Nevertheless, they found that the treatment was well tolerated with no side-effect differences [24].

In a small open-label study (n=19), Connor et al. investigated the use of guanfacine extended release (GXR) for the treatment of children and adolescents with symptoms related to traumatic stress. Subjects ranged from age 6 to 18 years old. Psychiatric comorbidities included ADHD (89.5%), PTSD (68.4%), separation anxiety disorder (10.6%), and reactive attachment disorder (5.3%). Those receiving GXR were given 1.0–4.0 mg at night. Connor et al. found that those in the GXR group experienced a significant improvement in PTSD symptoms, as measured by the UCLA Posttraumatic Stress Disorder Reaction Index. Adverse effects were mild and included sedation, headache, dry mouth, dizziness, and decreased diastolic blood pressure [25]. Unlike the previous findings in adults with PTSD only, patients with ADHD and PTSD comorbidity may have a preferential response. This preparation of guanfacine is approved for childhood ADHD as a monotherapy and as an adjunct to stimulant therapy.

The negative evidence base for guanfacine far outweighs that of clonidine. The more stringent guanfacine studies show no effect on PTSD and some intolerability. However, the smaller trial in comorbid children does show evidence for its efficacy. Of note, 12 of the 17 subjects (71%) in the Connor et al. study chose to continue with GXR treatment [25]. Given the relative lack of serious adverse effects, it may be reasonable to consider the use of guanfacine in specific patients or to extrapolate these findings and use this drug clinically in those adults with both ADHD and PTSD.

Although there have been no major trials showing the efficacy of the alpha-2 receptor agonists for the treatment of PTSD, there have been numerous case reports supporting their use. Porter and Bell described a case in which the use of clonidine was associated with a decrease in symptoms of reenactment, aggression, and nightmares in an 11-year-old girl with PTSD. This patient was started on 0.05 mg of clonidine three times a day, which resulted in a subjective improvement of her symptoms. Symptoms continued to improve with an increase to 0.1 mg of clonidine three times daily as well. When pharmacotherapy was disrupted, her aggression, inappropriate sexual behavior, and nightmares quickly resumed. Shortly after restarting her prior dose of clonidine, the symptoms once again improved [26].

In 2012, Alao et al. published two case reports showing the successful use of clonidine as a treatment for nightmares specifically in combat veterans with PTSD. The first case described a 48-year-old man who fought in the Bosnian war for 15 months. He presented with symptoms of depression, flashbacks, exaggerated startle response, and nightmares. Treatment with venlafaxine ER and olanzapine provided no relief. After 2 weeks of daily treatment with 0.1 mg of clonidine, the patient reported an improvement in his nightmares and sleep quality. The dose was later increased to 0.1 mg twice daily and olanzapine was discontinued. For 1 year of follow-up, his nightmares and other PTSD symptoms continued to be well controlled on this regimen [27].

The second case published by Alao et al. described a 33-year-old patient who fought in Iraq and Afghanistan. He presented with nightmares, flashbacks, exaggerated startle response, and avoidant behavior. He also had comorbid traumatic brain injury that manifested as short-term memory loss, headaches, dizziness, and hearing impairment. He was initially treated with cognitive therapy, citalopram, clonazepam, and prazosin. After no improvement in his nightmares, prazosin was discontinued, and the patient was started on 0.1 mg of clonidine with titration of the dose to 0.3 mg. After 2 weeks of treatment, he reported a marked improvement in his nightmares [27].

## Summary

PTSD remains a difficult anxiety disorder to treat, as the FDA-approved SSRIs may lead to response but not a full remission of symptoms. Insomnia and agitation are often residual and may go untreated. BZD sedatives can be helpful, but with the elevated addiction risk in PTSD, they are often avoided or used after failure of other nonaddictive, off-label treatments. Drugs with antihistaminergic properties, such as hydroxyzine and quetiapine, are often used clinically. As the evidence supporting the use of the alpha-1 receptor agonist antihypertensives for nightmares has mounted, there seems to be escalating interest in the use of the alpha-2 receptor agonist antihypertensive agents that also manipulate and dampen CNS’ noradrenergic neurocircuitry. This review suggests that there is a very reasonable theoretical rationale to use the alpha-2 receptor agonists in practice, but the evidence base is still quite limited. Clonidine seems to be preferred in practice perhaps because it is more widely known or in that its nonspecificity for alpha-2 receptor subtypes does make it more sedating outright. Clinicians may actually view these “side effects” as desirable, as they may induce sleep and relaxation effects in agitated PTSD patients. GXR likely has the most robust data, albeit in children with concurrent ADHD and PTSD. Either agent might be considered when residual PTSD agitation and hyperarousal symptoms exist and SSRIs fail. More importantly, these agents might be considered when the addiction risk of BZD use is too high, or if use of atypical antipsychotics is too risky due to metabolic or neuromuscular side effects. Although there are no studies directly comparing the side effects of alpha-2 receptor agonists with those of BZD and atypical antipsychotics, the alpha-2 receptor agonists seem safer as they do not induce permanent movement disorder, cause metabolic syndrome, or foster dependence or addiction.

## Abbreviations:

ADHD: attention deficit hyperactivity disorder
APA: American Psychiatric Association
AUD: alcohol use disorder
BZD: benzodiazepine
CAPS: Clinician-Administered PTSD Scale
CNS: central nervous system
CSF: cerebrospinal fluid
EPS: extrapyramidal symptoms
FDA: Food and Drug Administration
GXR: guanfacine extended release
IES-R: Impact of Event Scale-Revised
MAOI: monoamine oxidase inhibitor
MDD: major depressive disorder
NE: norepinephrine
PTSD: posttraumatic stress disorder
SCL-90-R: Symptom Checklist-90-Revised
SNS: sympathetic nervous system
SSRI: selective serotonin reuptake inhibitors
TCA: tricyclic antidepressant
TD: tardive dyskinesia
